# Somatic *MED12* mutations are associated with poor prognosis markers in chronic lymphocytic leukemia

**DOI:** 10.18632/oncotarget.2753

**Published:** 2014-12-18

**Authors:** Kati Kämpjärvi, Tiina M. Järvinen, Tuomas Heikkinen, Amy S. Ruppert, Leigha Senter, Kevin W. Hoag, Olli Dufva, Mika Kontro, Laura Rassenti, Erin Hertlein, Thomas J. Kipps, Kimmo Porkka, John C. Byrd, Albert de la Chapelle, Pia Vahteristo

**Affiliations:** ^1^ Department of Medical Genetics and Genome-Scale Biology Research Program, University of Helsinki, Helsinki, Finland; ^2^ Department of Molecular Virology, Immunology and Medical Genetics, Division of Human Cancer Genetics, Comprehensive Cancer Center at The Ohio State University, Columbus, OH, USA; ^3^ Department of Internal Medicine, Division of Hematology, Comprehensive Cancer Center at The Ohio State University, Columbus, OH, USA; ^4^ Hematology Research Unit Helsinki, Department of Medicine, Helsinki University Central Hospital and University of Helsinki, Helsinki, Finland; ^5^ Moores University of California-San Diego Cancer Center, University of California San Diego, CA, USA

**Keywords:** Chronic lymphocytic leukemia, MED12, somatic mutation, cancer genetics, prognosis

## Abstract

Chronic lymphocytic leukemia (CLL) is the most common leukemia in adults. We performed systematic database search and identified highly specific *MED12* mutations in CLL patients. To study this further, we collected three independent sample series comprising over 700 CLL samples and screened *MED12* exons 1 and 2 by direct sequencing. Mutations were identified at significant frequency in all three series with a combined mutation frequency of 5.2% (37/709). Positive mutation status was found to be associated with unmutated IGHV and ZAP70 expression, which are well-known poor prognosis markers in CLL. Our results recognize CLL as the first extrauterine cancer type where 5′ terminus of *MED12* is mutated at significant frequency. Functional analyses have shown that these mutations lead to dissociation of Cyclin C-CDK8/19 from the core Mediator and to the loss of Mediator-associated CDK kinase activity. Additional studies on the role of *MED12* mutation status as a putative prognostic factor as well as mutations' exact tumorigenic mechanism in CLL are warranted.

## INTRODUCTION

Chronic lymphocytic leukemia (CLL) is the most prevalent adult leukemia in Western societies. The disease is characterized by the accumulation of monoclonal CD5-, CD19-, and CD23-positive B cells in the blood and lymphoid tissues. The clinical course of patients with this disease is heterogeneous, ranging from highly indolent, which may never require therapy, to relatively aggressive, which may require therapy soon after diagnosis. CLL can be divided into two subclasses based on the occurrence of somatic hypermutation in the immunoglobulin heavy-chain variable (IGHV) genes. [1] The subclass with mutated IGHV genes (M-CLL) has an improved treatment-free and overall survival compared to the subclass with unmutated IGHV genes (U-CLL) [2, 3]. Currently, there is no curative drug therapy for CLL, but chemo-immunotherapy can significantly improve survival [4]. As the course of the disease is highly unpredictable, there is an urgent need to better elucidate the biology of CLL.

Recent large-scale sequencing studies have shown that the average number of somatic mutations in CLL is smaller than in solid tumors but comparable with other hematological neoplasms [5]. Only a few genes (e.g. *TP53*, *NOTCH1, SF3B1, XPO1, BIRC3, MYD88*) have emerged as frequently mutated in CLL. In contrast, many other genes are recurrently mutated at lower frequencies (2–5%). There are also some CLL cases which do not have mutations in any known CLL driver gene. [Reviewed in 6] These observations emphasize the molecular heterogeneity of the disease and highlight the need for a comprehensive classification of CLL based on molecular characteristics.

Mediator is a multisubunit protein complex required for activator-dependent transcription of most protein coding genes. Mutations or altered expression of many Mediator subunits have been associated with various medical conditions ranging from cancer to neuropsychiatric illnesses. *Mediator complex subunit 12* (*MED12*), a 45 exon gene on Xq13.1, encodes a subunit of the Mediator's kinase module. *MED12* was first implicated in human tumorigenesis when highly specific exon 2 mutations were identified in as many as 70% of uterine leiomyomas [7]. Functional analyses indicated that these mutations disrupt the interaction between MED12 and Cyclin C-CDK8 and lead to a diminished Mediator-associated CDK kinase activity [8]. Recently, we identified mutations also in exon 1 and showed by expression profiling and functional analyses that they lead to similar unique global gene expression pattern and shared tumorigenic mechanisms as exon 2 mutations [8, 9].

Following the observation of specific *MED12* mutations in myomas, similar mutations have been searched for in several tumor types [e.g. 10–14]. Mutations have been detected at significant frequency in breast fibroadenomas (59%) and uterine leiomyosarcomas (7–20%), and rarely in extrauterine leiomyomas and leiomyosarcomas, endometrial polyps, and colorectal cancer. To analyze this more thoroughly, we performed a systematic database search utilizing the Catalogue of Somatic Mutations in Cancer (COSMIC) [15]. Interestingly, the search revealed five *MED12* mutations in CLL. All these mutations were in exon 1 or 2: three affected the mutation hotspots observed in uterine leiomyomas (codons L36 and G44), the fourth was a missense change altering the last amino acid of exon 1 (E33K), and the fifth resulted in A59P substitution. These data have been retrieved from various recent exome and whole genome sequencing studies, and no mutations in other parts of the gene have been identified. This surprising database finding prompted us to systematically study the contribution of *MED12* in CLL.

## RESULTS

Altogether 746 samples from three independent sample series were included in the study. *MED12* exons 1 and 2 were successfully sequenced in 709 samples (709/746, 95%). This analysis revealed mutations in 37 samples (37/709; 5.2% Supplementary Table 2; two samples had two mutations). Mutations were recurrently identified in each sample series with frequencies of 7.7% (20/260; CRC samples), 3.6% (10/274; Sample Bank samples), and 4.0% (7/175; Finnish samples). There is no obvious bias in the collection or characteristics of the sample series, which could explain the slightly higher mutation frequency in the CRC series.

**Figure 1:**
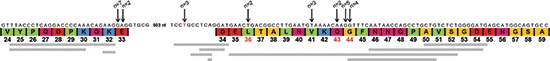
Schematic presentation of *MED12* exon 1 and exon 2 mutations in CLL identified in this study Amino acids and codon numbers are shown below the DNA sequence. Arrows indicate substitutions and grey bars insertion/deletion mutations, respectively. Numbers above arrows indicate how many times each mutation was detected. Mutation hotspot codons observed in uterine leiomyomas are marked with red. Amino acids are color-coded according to their side-chain pK_a_s and charge at physiological pH 7.4. Red = negatively charged; green = hydrophobic; yellow = small non-polar, magenta = polar, blue = positively charged.

All the observed mutations were either missense mutations affecting highly conserved amino acids or small insertions or deletions resulting in an in-frame transcript (Figure 1). The most commonly mutated sites were codons G44 (11/39; 28%, including 9 missense mutations and 2 deletions) and E33 (9/39; 23%, all missense mutations). Many of the remaining mutations affected the previously reported mutation hotspots (c.100-8T, L36, and Q43). Two variants with unknown significance, a substitution c.1–28G > T in the UTR and a silent p.R16R in exon 1, were also observed. As their effect on protein function is unclear, they were excluded from the mutation frequency calculations and the association analyses. Detailed information on the observed mutations and the mutation positive samples are presented in Supplementary Table 2.

Association analyses were performed based on the molecular and clinical data available from the patients in the CRC series. Interestingly, *MED12* mutations were associated with markers of poor prognosis: unmutated IGHV [2, 3] (*p* = 6.8×10^−4^), ZAP-70 protein positivity [16] (≥ 20% expression) (*p* = 7.1×10^−4^), and unmethylated (< 20%) ZAP-70 [17] (*p* = 5.5×10^−4^) (Table 1). More specifically, all 20 patients with the *MED12* mutation had the established poor prognostic unmutated IGHV disease status, and all 19 who were measured for similar adverse prognostic ZAP-70 methylation were unmethylated. No differences in time to treatment or overall survival were, however, detected.

**Table 1:** Demographic and molecular features by *MED12* mutation status in CLL Research Consortium sample series *MED12* mutations are associated with markers of poor prognosis in CLL: unmutated IGHV, ZAP-70 protein positivity, and unmethylated ZAP-70.

CLL Marker Data	*MED12* Not Mutated (*n* = 240)	*MED12* Mutated (*n* = 20)	*P*
Age			
Median	55	56	0.55
Range	26–82	44–72	
Sex			
Male	169 (70%)	16 (80%)	0.45
Female	71 (30%)	4 (20%)	
IGHV			
Mutated	79 (33%)	0 (0%)	**< 0.001**
Unmutated (≥ 98%*)	161 (67%)	20 (100%)	
CD38			
Negative	115 (48%)	5 (25%)	0.06
Positive (≥ 20%)	125 (52%)	15 (75%)	
ZAP-70			
Negative	117 (49%)	2 (10%)	**< 0.001**
Positive (≥ 20%)	123 (51%)	18 (90%)	
ZAP-70 Methylation			
Methylated (≥ 20%)	84 (35%)	0 (0%)	**< 0.001**
UnMethylated (< 20%)	153 (65%)	19 (100%)	
Unknown	3	1	

* Sequences showing 98% or greater homology to the corresponding germ-line IGHV sequence were considered unmutated.

## DISCUSSION

Highly specific *MED12* mutations were first observed in benign uterine leiomyomas [7]. Subsequent mutation analyses have revealed recurrent mutations also in breast fibroadenomas and uterine leiomyosarcomas [11–14]. Here, prompted by the systematic analysis of somatic mutation database, we have collected three independent sample series totaling a comprehensive series of more than 700 CLL samples. Mutation screening revealed recurrent mutations in all three series with a combined mutation frequency of 5.2% (37/709). These results indicate that in addition to solid hormone dependent female tumors, *MED12* mutations are involved also in CLL pathogenesis. Significant mutation frequency also implies that *MED12* should be recognized as an important cancer gene in CLL.

As in uterine leiomyomas, leiomyosarcomas, and breast fibroadenomas, all *MED12* mutations in CLL are missense changes or small in-frame insertions and deletions. The most commonly affected mutation hotspot in all tumor types is codon G44. Despite these similarities in mutation profiles, also an interesting difference was observed. A novel *MED12* mutation hotspot was identified in CLL as mutations affecting the last amino acid of exon 1, E33, were seen nine times (Figure 1 and Supplementary Table 2). Similar mutations have previously been observed twice in CLL [18], once in bladder carcinoma [15], and twice in colorectal cancer [15, 19]. No missense mutations at E33 have been reported in uterine leiomyomas, although a few deletions removing the site have been detected [9]. Altogether 12 of the 39 mutations observed in this study were in exon 1 (31%) and 27 (69%) in exon 2. The location of mutations in CLL (current study and COSMIC database) is statistically different from the location of mutations in uterine leiomyomas, where nearly all reported mutations (173/178; 97%) have been in exon 2 (*p* = 6.0×10^−7^) [7, 9, 20]. Our recent data shows that the Cyclin C-CDK8 binding domain of MED12 consists of its 100 N-terminal amino acids, largely encoded by exons 1 and 2 [8]. Gene expression profiling and functional analyses indicate that mutations within these exons lead to similar expression profiles and shared tumorigenic mechanism, involving diminished Mediator-associated kinase activity [8, 9]. Whether the observation of different exon1 and 2 mutation ratios reflects true tissue specific functional relevance will need to be determined with further research.

Extensive molecular and clinical data were available from one US sample series. Interestingly, association analyses showed significant association between *MED12* mutations and unmutated IGHV status, ZAP70 expression, and unmethylated ZAP70, which are well-known poor prognosis markers in CLL [2, 3, 16, 17]. Possible associations with time to treatment and overall survival were also analyzed, but no differences were observed. The role of *MED12* mutational status as a putative prognostic factor will need to be studied further, where our data support investigations in a larger series of high-risk patients adequately powered to detect moderate differences in clinical outcome.

A few *MED12* mutations in CLL have been identified in recent large-scale sequencing studies [18, 21–24]. As these have been rare mutation positive cases in independent studies, the significant *MED12* mutation frequency in CLL has not been recognized prior to this study. Also our literature search of PubMed with the words “*MED12”* and “leukemia” did not produce any results. This highlights the utility of comprehensive mutation databases, which facilitate identification of true cancer genes which are mutated recurrently but at moderate frequency.

Our results recognize CLL as the first extrauterine cancer type where the 5′ terminus of *MED12* is mutated at significant frequency. We also show that these mutations are associated with poor prognosis markers in CLL. Identification of such specific mutations in highly different tumor types -benign uterine leiomyomas and breast fibroadenomas, and a malignant chronic leukemia- is both puzzling and intriguing. While the immediate effects of the mutations on the interactions and kinase activity of the Mediator complex may be similar, the downstream effects on transcription may differ in various tissue types. Overall, the results from this study suggest that *MED12* plays a role in the pathogenesis of CLL. Further investigations of *MED12*'s function in CLL are warranted.

## METHODS

Three independent sample series from the United States and Finland comprising altogether 746 samples were collected for the study. The first US series consisted of 278 samples obtained from the CLL Research Consortium (CRC) and the second included 292 samples from The Ohio State University's Human Genetics Sample Bank. The Finnish CLL series, consisting of 176 samples, was obtained from the Helsinki University Central Hospital clinical sample collection. Samples were collected either after signed informed consent (living patients) or with authorization from the Ethics Committee of the Helsinki University Central Hospital (deceased Finnish patients). The study was approved by the Institutional Review Board of each participating CRC site, The Ohio State University, and the Ethics Review Board of the Hospital District of Helsinki and Uusimaa, Helsinki, Finland.

All *MED12* mutations observed in exome or whole genome sequencing studies on CLL have resided in exons 1 and 2. Even though some mutations in other exons might be detectable by targeted sequencing with higher depth of coverage, results from these studies suggest that similarly to uterine leiomyomas, *MED12* mutations in CLL are restricted to exons 1 and 2. In this study, mutation screening of exons 1 and 2 was done by direct sequencing using previously described primers [9, 12]. Samples were sequenced with Applied Biosystems 3730 DNA Analyzer (Life Technologies, Thermo Fischer Scientific Waltham, MA, USA) and analyzed manually and with Mutation Surveyor (Softgenetics, State College, PA, USA) or Lasergene SeqMan Pro (DNASTAR, Madison, WI, USA) softwares. See Supplemental Data for more details on the sample series and methods.

Molecular and clinical data were available from patients in the CRC series. Associations between the *MED12* mutations and various demographic and molecular characteristics were analyzed using Fisher's exact and Wilcoxon rank sum tests for categorical and continuous variables, respectively.

## SUPPLEMENTARY DATA AND REFERENCES




