# Supplementary material for: Somatic MED12 mutations are associated with poor prognosis markers in chronic lymphocytic leukemia
Source: Oncotarget. 2014 Dec 18;6(3):1884–8. doi: 10.18632/oncotarget.2753 (PMC4359339; doi:10.18632/oncotarget.2753)
Supplement: Supplementary file 1 [file oncotarget-06-1884-s001.pdf]

SUPPLEMENTARY DATA

Patient series

Three independent CLL patient series, two from the United States and one from Finland, were included in the study. The total number of patients was 746.

Chronic Lymphocytic Leukemia Research Consortium (CRC) sample series

Frozen peripheral-blood mononuclear cells of 278 CLL patients across the USA were obtained through the CLL Research Consortium (CRC; http://cll.ucsd.edu/), a multi-institutional program project investigating new molecular targets that have potential to lead to the development of novel and effective treatments for CLL. The CRC Tissue Core Biorepository processes and banks biospecimens according to established standardized operating procedures. A multiparameter flow cytometry is used to determine the CLL cell surface antigen phenotype. In addition, the expression of ZAP-70 and the IGHV mutation status is determined on all samples received. A more detailed description of the CRC cohort is given in Rassenti et al., 2004 and 2008 [1, 2]. All studies are performed with the patient's informed consent that is approved by the Institutional Review Board of each participating CRC study site, and in accordance with the Declaration of Helsinki.

The Ohio State University's Human Genetics Sample Bank sample series

DNA samples from 292 CLL cases were obtained from the Human Genetics Sample Bank at The Ohio State University (OSU), Columbus, OH, USA (http://internalmedicine.osu.edu/genetics/research/human-genetics-sample-bank/). The samples included in this study were collected as part of ongoing studies approved by the Institutional Review Board at OSU. All patients gave informed consent for the research use of their specimens, in accordance with the Declaration of Helsinki.

Helsinki University Central Hospital clinical sample series

Finnish sample series was collected under the authority of a research project at the Helsinki University Central Hospital and the Hematology Research Unit at University of Helsinki investigating molecular pathogenesis, predisposing factors, and individualized care of hematological diseases (HRUHLAB2; http://www.hematology.fi/hruhlab2). Patients diagnosed with CLL at the Hospital District of Helsinki and Uusimaa (HUS) were identified from the Finnish Hematological Registry and Biobank project (FHRB), compiling a list of altogether 259 patients. Current status of the patients was confirmed from their medical records and patients at terminal stage or with memory disorder (n = 6) were excluded from the study. Invitation to participate in the study was sent to altogether 194 living patients, of whom 179 (92%) signed an informed consent and 15 (8%) declined from the study or did not respond to the invitation. By signing the informed consent, patients permitted the usage of their previously derived blood/bone marrow samples and the collection of information from their medical records. Authorization to use previously collected samples and clinical information of deceased patients (n = 59) was obtained from the Ethics Committee of the Helsinki University Central Hospital. Samples from altogether 176 patients (129 living and 47 deceased) were found at the hospital's archives and included in the study. No new samples were collected for this study. General information on the sample series can be found in Supplementary Table 1.

DNA extraction

US samples

Genomic DNA of the CRC samples was extracted from cryopreserved peripheral-blood mononuclear cells and that of the OSU Human Genetics Sample Bank specimens from whole blood using a standard phenol-chloroform isolation method, and a traditional salting out procedure [3], respectively. DNA concentration and purity were measured with Nanodrop 1000 (for the CRC samples; Nanodrop Technologies, Wilmington, DE) and 2000 (for the Sample Bank samples; Thermo Fisher Scientific, Waltham, MA, USA).

Finnish samples

Genomic DNA was extracted from the archived cytogenetic preparations (fixed and stored in 3:1 methanol/acetic acid) with DNeasy® Blood and Tissue Kit (Qiagen, Hilden, Germany) according to manufacturer's instructions. DNA concentration and purity were measured with Nanodrop 8000 (Thermo Fisher Scientific).

MED12 mutation screening

MED12 exon 1 and 2 mutation status was determined by direct sequencing. Previously described primers for exon 1 and primers amplifying all the observed mutation hotspots and insertion/deletions in exon 2 were used in the screening [4, 5]. PCR conditions are available upon request. The US samples were sequenced with Applied Biosystems 3730 DNA Analyzer (Life Technologies, Thermo Fischer Scientific) at the OSU Comprehensive Cancer Center's Nucleic Acid

Shared Resource (Columbus, OH, USA). Sequencing traces were analyzed manually and with Mutation Surveyor (Softgenetics, State College, PA, USA) or Lasergene SeqMan Pro (DNASTAR, Madison, VI, USA) softwares. Sequencing of the Finnish samples was performed on an Applied Biosystems 3730 Automatic DNA Analyzer (Life Technologies, Thermo Fischer Scientific) at Institute for Molecular Medicine Finland (FIMM), Genome and Technology Centre, Helsinki, Finland. The sequences were analyzed both manually and with Mutation Surveyor software.

SUPPLEMENTARY REFERENCES

1. Rassenti LZ, Huynh L, Toy TL, Chen L, Keating MJ, Gribben JG, Neuberg DS, Flinn IW, Rai KR, Byrd JC, Kay NE, Greaves A, Weiss A, et al. ZAP-70 compared with immunoglobulin heavy-chain gene mutation status as a predictor of disease progression in chronic lymphocytic leukemia. N Engl J Med. 2004; 351:893–901.
2. Rassenti LZ, Jain S, Keating MJ , Wierda WG, Grever MR, Byrd JC, Kay NE, Brown JR, Gribben JG, Neuberg DS, He F, Greaves AW, Rai KR, et al. Relative value of ZAP-70, CD38, and immunoglobulin mutation status in predicting aggressive disease in chronic lymphocytic leukemia. Blood. 2008; 112:1923–1930.
3. Miller SA, Dykes DD, Polesky HF, . A simple salting out procedure for extracting DNA from human nucleated cells. Nucleic Acids Res. 1988; 16:1215.
4. Kämpjärvi K, Mäkinen N, Kilpivaara O, Arola J, Heinonen HR, Böhm J, Abdel-Wahab O, Lehtonen HJ, Pelttari LM, Mehine M, Schrewe H, Nevanlinna H, Levine RL, et al. Somatic MED12 mutations in uterine leiomyosarcoma and colorectal cancer. Br J Cancer. 2012; 107:1761–1765.
5. Kämpjärvi K, Park MJ, Mehine M, Kim NH, Clark AD, Bützow R, Böhling T, Böhm J, Mecklin JP, Jävinen H, Tomlinson IP, van der Spuy ZM, Sjoberg J, et al. Mutations in exon 1 highlight the role of MED12 in uterine leiomyomas. Hum Mutat. 2014; 35:1136–1141.

SUPPLEMENTARY FIGURE AND TABLES

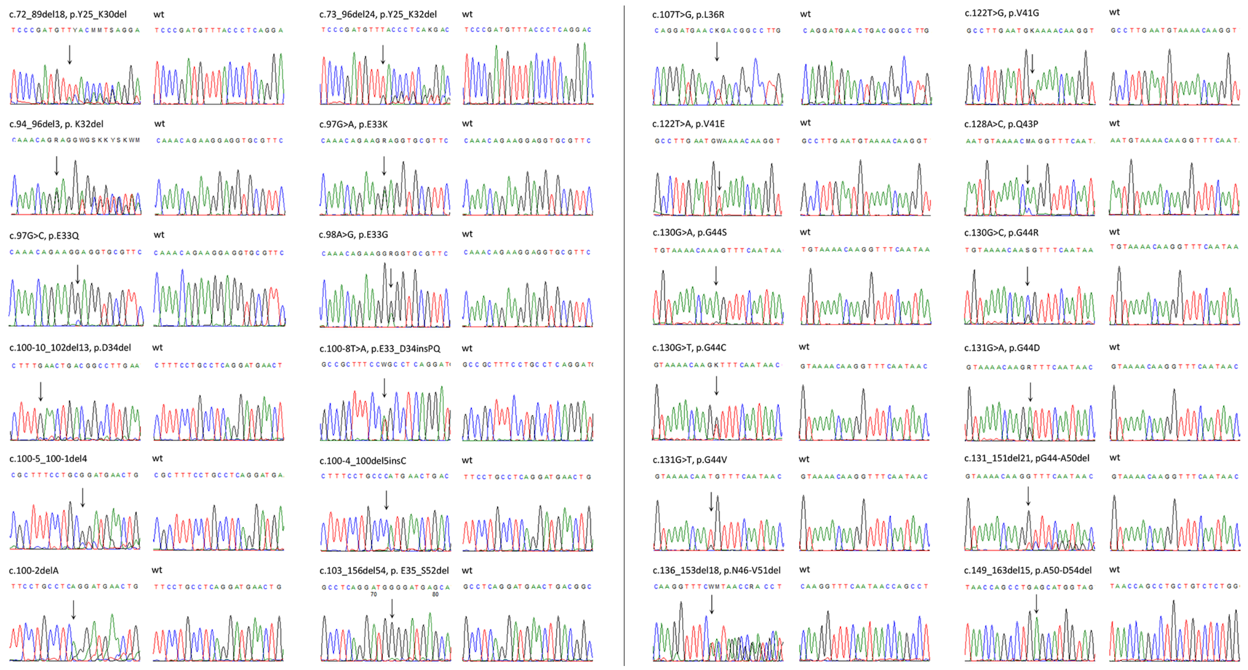

Supplementary Figure 1: Sequence chromatograms of *MED12* exon 1 and 2 mutations detected in CLL samples. The CLL sample with a mutation is shown on the left and the wild type reference sequence on the right. Arrows indicate the mutation sites; the exact position of the missense mutations and the start sites of the insertion/deletion mutations. If the same mutation was observed several times, only one representative figure is shown.

Supplementary Table 1: CLL sample series included in the study

Sample series	N	Sex (male/female/n.a.)	Mean age at diagnosis	Sample procurement relative to treatment before/after/n.a.)
Ohio Sample Bank (USA)	292	179/108/5	56	114/178/-
CRC (USA)	278	196/82/-	56	189/60/29
HUCH (Finland)	176	110/66/-	59	134/20/22

n.a.: Not available

Supplementary Table 2: *MED12* exon 1 and 2 mutations observed in this study and the clinical features of the mutation positive samples.
